# Whither structural biologists?^1^

**DOI:** 10.1107/S2052252522005802

**Published:** 2022-06-08

**Authors:** Gerard J. Kleywegt, Sameer Velankar

**Affiliations:** a European Molecular Biology Laboratory, European Bioinformatics Institute, Wellcome Genome Campus, Hinxton, Cambridge CB10 1SD, United Kingdom

**Keywords:** structural biology, AlphaFold, protein-structure prediction, career perspectives, data-driven revolution, editorial

## Abstract

The scientific impact of accurate protein-structure prediction methods is being felt already, but how might they affect the work and careers of structural biologists?

Between December 2020 and July 2021, several spectacular developments in the field of protein-structure prediction changed structural biology profoundly, and they are expected to have an impact on much of modern (molecular) biology, medicine, biochemistry and biotechnology. The unprecedented accuracy of blind protein-structure predictions produced by DeepMind’s *AlphaFold2* was revealed at the CASP 14 meeting in December 2020. In July 2021, this was followed by publication of the method and release of the code (Jumper *et al.*, 2021 ▸). Simultaneously, a prediction method from the Baker lab that achieved similar accuracy was published (Baek *et al.*, 2021 ▸). A week later, an additional publication described proteome-scale application of protein-structure prediction using *AlphaFold2*. This coincided with the launch of a new resource in which DeepMind and EMBL–EBI collaborate to make hundreds of thousands (and, eventually, hundreds of millions) of high-quality predicted protein structures openly available: the AlphaFold Protein Structure Database, or AlphaFold DB (Tunyasuvunakool *et al.*, 2021 ▸; Varadi *et al.*, 2022 ▸). These developments stunned not only the protein-structure prediction community, but most of the structural biology, bioinformatics, and machine-learning communities and beyond. It was truly an ‘*annus mirabilis*’.

Much has been said and written about these developments in the scientific, technical and popular press (not to mention on social media) and the journal *Nature Methods* selected protein-structure prediction as its *Method of the Year* (Editorial, 2022 ▸). The potential impact of the developments has also been the subject of much prognostication and speculation. When AlphaFold DB was first announced, a group of senior EMBL structural biologists released a white paper discussing its possible impact (https://www.embl.org/news/science/alphafold-potential-impacts/). At the time of writing (late May 2022), using the term ‘alphafold’ to search the literature at Europe PMC (https://europepmc.org/search?query=alphafold), there are over 700 published research papers, over 150 unpublished preprints and, somewhat astonishingly, almost 300 review papers.

The structural community was impressively quick to embrace the new resources (both the software and the database) and a flurry of preprints appeared in bioRxiv within weeks, both analysing the holdings of the database (*e.g.* correlating the estimated reliability of models and the location of intrinsically disordered regions in proteins) and putting the software through its paces (*e.g.* various ‘hacks’ to make *AlphaFold2* predict structures of multimeric complexes). Experimental structural biologists were also quick to react and within days of AlphaFold DB being announced and the AlphaFold code being released, there were many reports of structures (X-ray and cryo-EM mostly) that had previously resisted solution but that suddenly could be cracked using one or more (parts of) AlphaFold (DB) models. Methods and software developers were also quick to adapt their procedures and programs to make use of the astonishing new opportunities offered by high-quality protein-structure predictions. Over time, scientists outside the structural field are expected to embrace and discover the power of the new methods and the fact that soon a structure model will be available (or easily obtainable) for just about any protein sequence, be it natural or designed. Moreover, developments in the structure-prediction field will continue, *e.g.* concerning structure prediction for macromolecular and ligand complexes, RNA, post-translational modifications, and the impact of point mutations. The full scientific impact will take many years to be realized, and by then the new tools (methods and models) will be textbook material for undergraduate curricula.

While this is all fantastic news for bioscience, medicine and biotechnology, there are also concerns, not least in the field of structural biology, regarding the impact of these developments on funding and careers. We understand that when the announcement of AlphaFold DB was made via the CCP4 mailing list, there was a crystallography course on-going, and the news made some of the students on the course question their study or career choices. We wouldn’t be surprised if, upon first hearing the announcements, more than a few structural biologists went through several of the five stages of grief (denial, anger, bargaining, depression and acceptance) and have asked themselves questions such as: ‘*Is there even a need for experimental structure determination anymore?*’, ‘*Is there a future career in structural biology for me?*’, ‘*Will it be possible to get tenure in this field?*’, *‘Will funding agencies now think that structure determination is not necessary and stop funding my projects/methods development efforts?*’, ‘*Will expensive infrastructure (synchrotrons, microscopes, spectrometers) still get funded?*’.

It is almost unavoidable that there will be changes in terms of funding and career opportunities. However, as the adage (attributed to Einstein) goes: ‘*In the midst of every crisis, lies great opportunity*.’ Perhaps this time of profound change in our field is merely a transition to a new golden era for structural biology. For one thing, structure prediction still has limitations and is anyway not a panacea; also, predictions will need validation. More importantly, there will be entirely new opportunities leading to a ‘new normal’ in structural biology which will, in turn, support a new normal in biology. This means that there will continue to be a need for experimental structure determination (and the requisite people and infrastructure). However, it is likely that structures can generally be determined much more quickly than before and hence that we can focus more on the real objective of structural biology: to understand or influence function, mechanism, activity, binding interactions, *etc*. using structural information (as well as other biophysical and computational methods). Moreover, in the wider field of biology, medicine, biotechnology, agriculture *etc*., there will be a huge need for scientists (and teachers) who know and understand structures, who can assess which parts of a structure, be it experimental or predicted, are likely to be reliable enough to (help) answer the biological question that is posed, who can assess which prior biological data about function *etc*. can be explained in light of a structure, who can compare multiple related structures and draw sensible conclusions, and who can help design follow-up experiments based on the insights gained from structures.

In summary, we are optimistic that, far from witnessing the end of structural biology, we are part of an exciting revolution in biology where structure will play a much more prominent role than in the past, at least on a par with the role that protein sequences are playing today.
